# An automated screening method for detecting compounds with goitrogenic activity using transgenic zebrafish embryos

**DOI:** 10.1371/journal.pone.0203087

**Published:** 2018-08-29

**Authors:** Sergio Jarque, Eva Fetter, Wouter J. Veneman, Herman P. Spaink, Ravindra Peravali, Uwe Strähle, Stefan Scholz

**Affiliations:** 1 RECETOX, Faculty of Science, Masaryk University, Brno, Czech Republic; 2 Department of Bioanalytical Ecotoxicology, Helmholtz Centre for Environmental Research–UFZ, Leipzig, Germany; 3 Department of Animal Sciences and Health, Institute of Biology, Leiden University, Leiden, The Netherlands; 4 Institute of Toxicology and Genetics, Karlsruhe Institute of Technology, Karlsruhe, Germany; Deakin School of Medicine, AUSTRALIA

## Abstract

The knowledge on environmentally relevant chemicals that may interfere with thyroid signaling is scarce. Here, we present a method for the screening of goitrogens, compounds that disrupt the thyroid gland function, based on the automatic orientation of zebrafish in a glass capillary and a subsequent imaging of reporter gene fluorescence in the thyroid gland of embryos of the transgenic zebrafish line tg(tg:mCherry). The tg(tg:mCherry) reporter gene indicates a compensatory upregulation of thyroglobulin, the thyroid hormone precursor, in response to inhibition of thyroid hormone synthesis. Fish embryos were exposed to a negative control compound (3,4-dichloroaniline), or a concentration series of known goitrogenic compounds (resorcinol, methimazole, potassium perchlorate, 6-propyl-2-thiouracil, ethylenethiourea, phloroglucinol, pyrazole) with maximum exposure concentration selected based on mortality and/or solubility. Exposure to 3,4-dichloroaniline decreased the fluorescence signal. All goitrogenic compounds exhibited clear concentration-dependent inductions of reporter fluorescence 1.4 to 2.6 fold above control levels. Concentration-response modelling was used to calculate goitrogenic potencies based on EC_50_ values. The new automated method offers an efficient screening approach for goitrogenic activity.

## Introduction

Many environmental compounds have been reported to affect the endocrine system in animals and humans. Compounds with estrogenic or androgenic activities are relatively well described and various *in vitro* or *in vivo* screening assays have been developed for their identification [1]. However, less attention has been paid to substances that may disrupt thyroid signaling. Given its crucial role in various metabolic, behavioral and developmental processes, alterations in the thyroid pathway may lead to diverse adverse effects. For instance, in humans, thyroid metabolism dysregulation has been associated to brain developmental, neurodevelopmental and behavioral disorders, autoimmunity, hepatotoxicity or cancer progression [2–6]. In animals, developmental delay, inhibition of metamorphosis, liver dysfunction and alteration in the cardiac function represent some of the effects described [7–9].

With the aim to provide an efficient screening tool for the detection of compounds with thyroid disrupting activities, several *in vitro* assays targeting a specific mechanism of action such as receptor competitive binding assays, enzymatic inhibition assays and serum proteins binding assays (transthyretin and thyroxine binding globulin) have been developed [10–12]. Xenopus and zebrafish embryos have been suggested and used as a model to identify thyroid disruptors, particularly goitrogenic compounds [13, 14]. Particularly, zebrafish embryos provide a small scale experimental system that is amendable to high-throughput screening testing and is considered as an alternative to testing of (adult) animals [15]. Furthermore, the use of zebrafish stages up to 120 hpf is not protected by current (European) animal welfare directives and fish embryos are considered as alternatives to testing of (adult) animals [16]. In contrast to genuine *in vitro* cellular systems, they represent a complex organism system where the hypothalamus-pituitary-thyroid (HPT) feedback loop is already established and functional. Expression of the gene for thyroglobulin (tg), the thyroid hormone precursor, is observed in the thyroid primordium at 32 hpf and detected in the thyroid follicles at 55 hpf. Thyroid hormone (T4) immunostaining overlaps with the expression patterns of tg [17]. Additional genes encoding for essential proteins involved in thyroid hormone (TH) synthesis such as NIS symporter, the transporter that mediates iodide uptake into the follicle cells, are also expressed at 40 hpf. Therefore, it is suggested that the thyroid gland in zebrafish is differentiated at 55 hpf. In situ hybridisation experiments showed that physiological concentrations of T_4_ were able to decrease the expression of thyroid-stimulating hormone β subunit (tshb) in the majority of pituitary cells in zebrafish embryos at 4 dpf, providing evidence that the negative feedback regulation is already functional at this stage [18].

Available assays with zebrafish embryos target T_4_ hormone levels either directly [19], or indirectly by measuring the corresponding gene expression of enzymes involved in the TH synthesis [20]. The latter can also be demonstrated using the transgenic zebrafish line tg(tg:mCherry)[21]. This line harbors a construct of the tg promotor and the gene for red fluorescent reporter protein mCherry. Given the negative feedback mechanism that regulates TH synthesis, hyper- and hypothyroidism may be efficiently detected and measured by differences of intensity in the tg:mCherry fluorescence. The fluorescence signal is correlated with the expression of genes involved in the TH synthesis [22]. The transgenic strain allows detection of thyroid disruption without any sample preparation by observation of fluorescence using a microscope. In order to establish a medium to high-throughput analysis, we designed an automatic procedure that uses the VAST BioImager platform to position the embryos [23], and subsequent image analysis to measure the tg:mCherry signal in the thyroid gland. To our knowledge, this is the first automated method for the screening of goitrogens using transgenic zebrafish embryos.

## Materials and methods

### Chemicals

The following chemicals purchased from Sigma-Aldrich (Deisenhofen, Germany) were used for the exposure of zebrafish embryos: 3,4-dichloroaniline (3,4-DCA, purity ≥98%), *N*, *N’*-ethylenethiourea (purity ≥98%), methimazole (purity ≥99%), phloroglucinol (purity ≥99%), potassium perchlorate (purity ≥99%), 6-propyl-2-thiouracil (PTU, analytical standard grade), pyrazole (purity ≥98%), resorcinol (purity ≥99%). For CAS numbers see Table 1. Log D values were obtained from chemspider (http://www.chemspider.com).

**Table 1:** Compound characteristics and estimated effect parameters for mortality and tg:mcherry induction in zebrafish embryos. Effect concentrations are given in μM. The log D is given as an indicator of hydrophobicity including potential ionization of the compound). BMD20 = concentration at which a 20% increase of the tgmcherry fluorescence was observed. TDI–thyroid disruption index = LC50/EC50_tg:mCherry induction_, 3,4-DCA– 3,4-dichloroaniline.

Compound	CAS-RN	Log D (pH 7.4)	LC_50 (48–120 hpf)_	EC_50_ (μM)	EC_50_ SE (μM)	Slope	Maximum fold induction	BMD 20 (μM)	TDI	EC_50_ T4 reduction^a^ (μM)	TDI (T4)^a^
Ethylenethiourea	96-45-7	-0.52	78922	366	116	3.8	1.9	246	216	135	-
Methimazole	60-56-0	-0.11	28800^b^	279	104	3.3	2.1	186	103	290	75
Phloroglucinol	108-73-6	0.24	>1x10^5^	1096	756	0.89	2.1	252	443^c^	2700	32
Potassium perchlorate	7778-74-7	n/a	33100	137	146	0.69	2.5	37.6	241	2.5	6030
Propylthiouracil	51-52-5	0.34	3500	334	115	1.7	2.1	163	11	137	20
Pyrazole	288-13-1	0.43	42428	637	67.8	5.7	1.3	399	67	-	
Resorcinol	108-46-3	0.86	5197	3.4	1.6	0.78	2.1	0.663	1529	82	62
3,4-DCA	95-76-1	2.6	29.4	n/a^d^	n/a	n/a	n/a	n/a	-	-	-

^a^ Comparative data for reduction of thyroid hormone levels (immunostaining) were taken from Thienpont et al. [13]

^b^ data obtained from Fetter et al. [22]

^c^ The TDI was calculated using the predicted fish embryo baseline toxicity of 60590 μM, calculated using the log D and according to Klüver et al. [32].

^d^ No induction but repression of tg:mCherry fluorescence

### Zebrafish maintenance and exposure

tg(*tg*:mCherry) zebrafish strain (F8) provided by the University of Brussels [21] crossed with the UFZ-OBI strain (generation F12, established from a stock of a local breeder). In the subsequent generation individual homozygous transgenic fish were identified by crossing with wildtype and used to establish a homozygous strain. Fish were cultured at 26±1°C at a 14:10 h light: dark cycle in a recirculating tank system similar as described by Westerfield [24]. Fish were cultured and used according to German and European animal protection standards and fish culture was approved by the Government of Saxony (Landesdirektion Leipzig, Aktenzeichen 75–9185.64).

In order to avoid interference of test chemicals with early thyroid gland development, zebrafish embryos were cultured in exposure medium [25] until 48 hours post fertilization (hpf). Subsequently, they were exposed to seven known thyroid endocrine disrupters for 3 days (48 to 120 hpf). The non-goitrogenic compound 3,4-dichloroaniline, that is used as reference compound in the zebrafish acute embryo toxicity test [25] was used as negative control. Stock solutions of ethylenethiourea, methimazole, potassium perchlorate, 6-propyl-2-thiouracil, phloroglucinol, pyrazole and resorcinol were freshly prepared in exposure medium. No solvents were used. The range of concentrations for each compound was selected by considering their solubility, effect concentrations for survival (Table 1) and the thyroid disrupting concentrations reported in a previous study that analyzed T_4_ content (Thienpont et al., 2011). Five concentrations and one control were tested per compound and replicate. In the second replicate the range of concentration was adjusted to improve fitting of concentration-response curves. Stability of the exposure solutions was confirmed (S1 Fig) by comparison of UV/VIS spectra in the range of 200–400 nm, obtained with an EPOCH microplate reader with cuvette slot (BIOTEK, Bad Friedrichshall, Germany). Given the lack of appropriate spectral properties stability of exposure concentrations was not analysed for potassium perchlorate. Embryos were kept in the incubator at 26°C and a 14:10 hours light:dark cycle. The exposure was conducted in crystallisation dishes covered with watchmaker glasses with 30 embryos in 30 ml exposure medium (for exposure medium refer to [26]). Prior to analysis, embryos were anesthetised by adding a 6 g/L stock solution of tricaine (Sigma-Aldrich, 10 μl per 400 μl exposure medium) and 16 (methimazole, phloroglucinol, potassium perchlorate) or 24 (resorcinol, propylthiouracil, 3,4-DCA, ethylenthiourea) embryos embryos were transferred to 96 well plates with rectangular wells (GE Healthcare, Little Chalfont, UK) with one embryo per well. The number of transferred embryos was increased from 16 to 24 in later experiments to ensure that enough thyroid gland images would be available for image analysis.

### Assessment of toxicity

Fish embryo acute toxicity tests were performed similar as described previously [27], using 10 or 25 embryos per concentration and replicate exposed in crystallization dishes from 48 to 120 hpf. No exchange of exposure media was performed. Lethality was identified by coagulation, missing heartbeat, a non–detached tail and/or missing of somites [28]. Raw data for mortality and phenotypic assessment are provided as S1 Table. Concentration-response curves for mortality are shown in S2 Fig.

### Screening of tg(tg:mCherry) embryos

Embryos at 120 hpf transferred to a 96well plate were analysed using the VAST (vertebrate automated screening technology) BioImager platform (Union Biometrica, Geel, Belgium) in combination with the LP sampler (Union Biometrica, settings are given in S2 Table) and a Leica fluorescence microscope (Leica DM6B equipped with a Leica digital camera DFC 365FX, settings are given in S3 Table). The VAST platform allows the automatic loading and positioning of embryos (Pardo-Martin et al., 2010) and automatic external imaging using a microscope. Embryos were positioned ventrally toward the microscope objective. Per embryo a bright field image, an autofocus fluorescence image and a Z-stack of 9 images were obtained. The Z-stack was required in case of autofocus failure and manual selection of the focal plane. Fluorescence images were obtained with 400 ms of fluorescent light exposure at maximal light intensity and a gain of 1. Given that a few embryos were not detected or did not show expression of the reporter gene the number of finally obtained and analysed images was lower than the number of embryos loaded onto the plate. However, care was taken, that at least 10 images per concentration and replicates were assessed. To avoid a time bias during the analysis the sequence of wells for analysis was arranged in order to obtain a uniform distribution over the entire period of analysis.

### Image analysis

**Fig 1:**
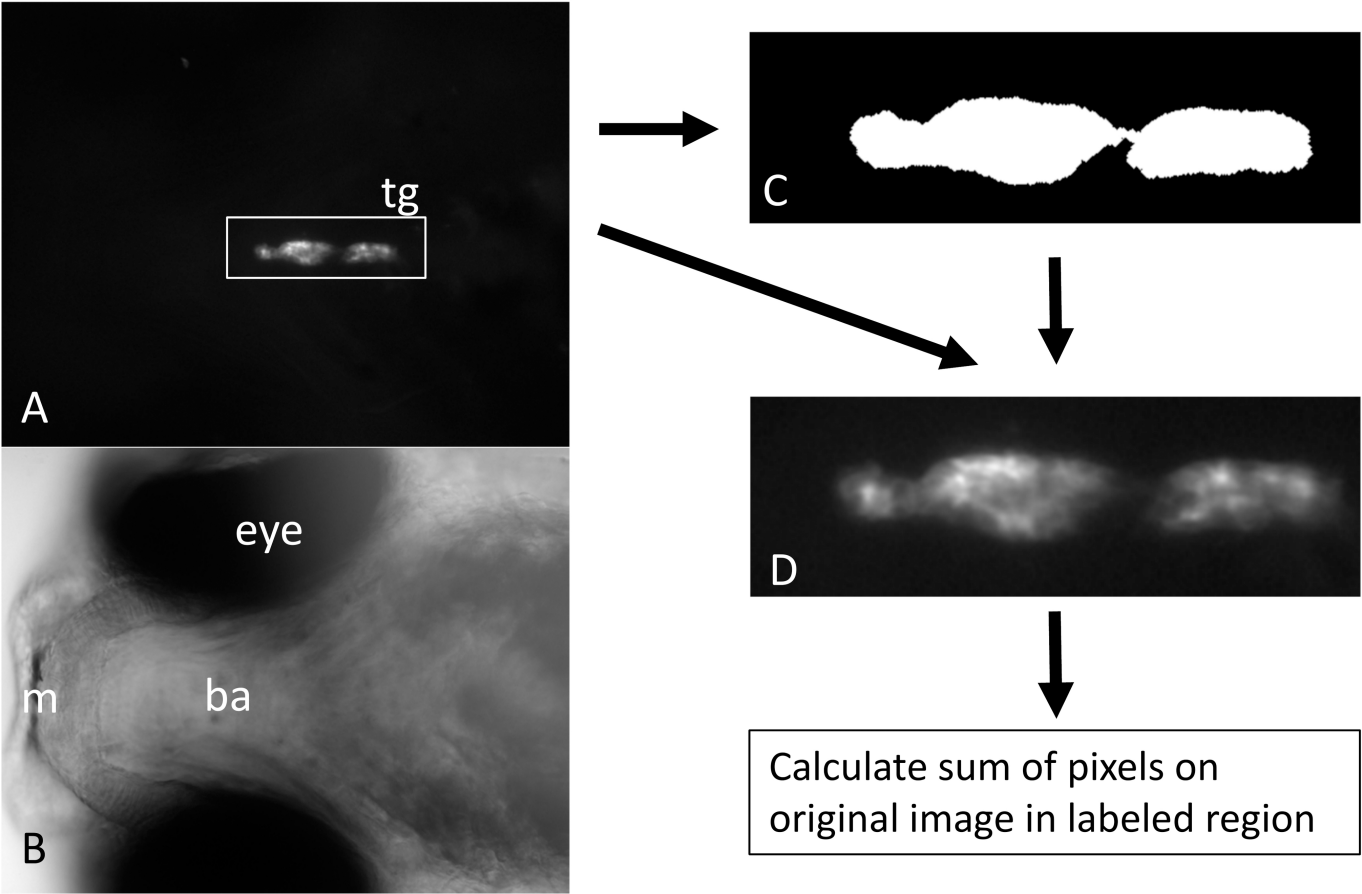
Overview of the KNIME workflow that was applied to quantify tg:mcherry fluorescence in transgenic embryos. The region of the thyroid gland (tg) was selected by manually drawing a rectangle on the appropriate autofocus or Z-stack images after background removal (A, corresponding bright field image shown in B). The thyroid gland was automatically detected and converted to a label, (C) The label was overlaid onto the original image (D) and used to calculate the sum of pixels. tg–area with thyroid follicles, ba–branchial arches, m–mouth.

The images were analysed using a KNIME workflow (Version 3.3.3., [29]) with the extension “KNIME image processing”. In brief, images were loaded into the workflow and the background was removed automatically using the “rolling ball” procedure of the image J plugin (rolling ball size set to 50 pixels). Subsequently the area containing the thyroid gland was manually selected by drawing a rectangle around the appropriate region using the interactive annotation node. This manual step was required to avoid interference with autofluorescence from pigment cells that occasionally were found close to the thyroid gland area. If possible the autofocus images were used. In case that the thyroid gland was out of focus due to the presence of autofluorescing pigment cells, an appropriate image from the Z-stack was selected. All subsequent steps were conducted without any user interaction. This included (1) identification of thyroid follicles using a threshold value of 20, (2) converting the binary threshold image into a label (3) extend the label and remove small spots outside the label and (4) overlay the label on the original image with removed background. Finally the sum of pixel (reflecting area of the thyroid gland and intensity of fluorescence) was calculated for the labeled area and used as a proxy of fluorescence intensity (see Fig 1 for an overview of the workflow). For each replicate the sum was of pixels was normalized by setting controls to the value of one. For data of individual embryos see S4 Table. The KNIME workflow used for image analysis is provided as supplement file (S1 Workflow).

### Statistical analysis

To characterize the potency of the investigated goitrogenic compounds, we calculated EC50 values of tg:mcherry fluorescence using the pixel sum obtained from image analysis. For each replicate the fluorescence intensity was normalized by dividing the mean fluorescence intensity of each concentration with the mean control fluorescence intensity. Concentration-response curves of tgmcherry fluorescence were fitted to the data using the Hill-slope equation (Eq 1) and used to estimate EC_50_ values.

$$y=Min+\frac{Max-Min}{1+{\left(\frac{x}{{LC}_{50}}\right)}^{-p}}$$ (1)

The parameter Min was set to 1 and the slope (p) and the maximum fluorescence (Max) were estimated. In case of resorcinol the highest test concentration was excluded from modelling due to decreasing tg:mCherry fluorescence. The software R and the package drc (R Core Team, 2015) embedded into a KNIME workflow were used to model concentration-response curves. The BMD20 (i.e. concentration with a 20% increase in fluorescence) was calculated using Eq (1) with y set to a value of 1.2.

## Results and discussion

### Screening of goitrogenic potencies

**Fig 2:**
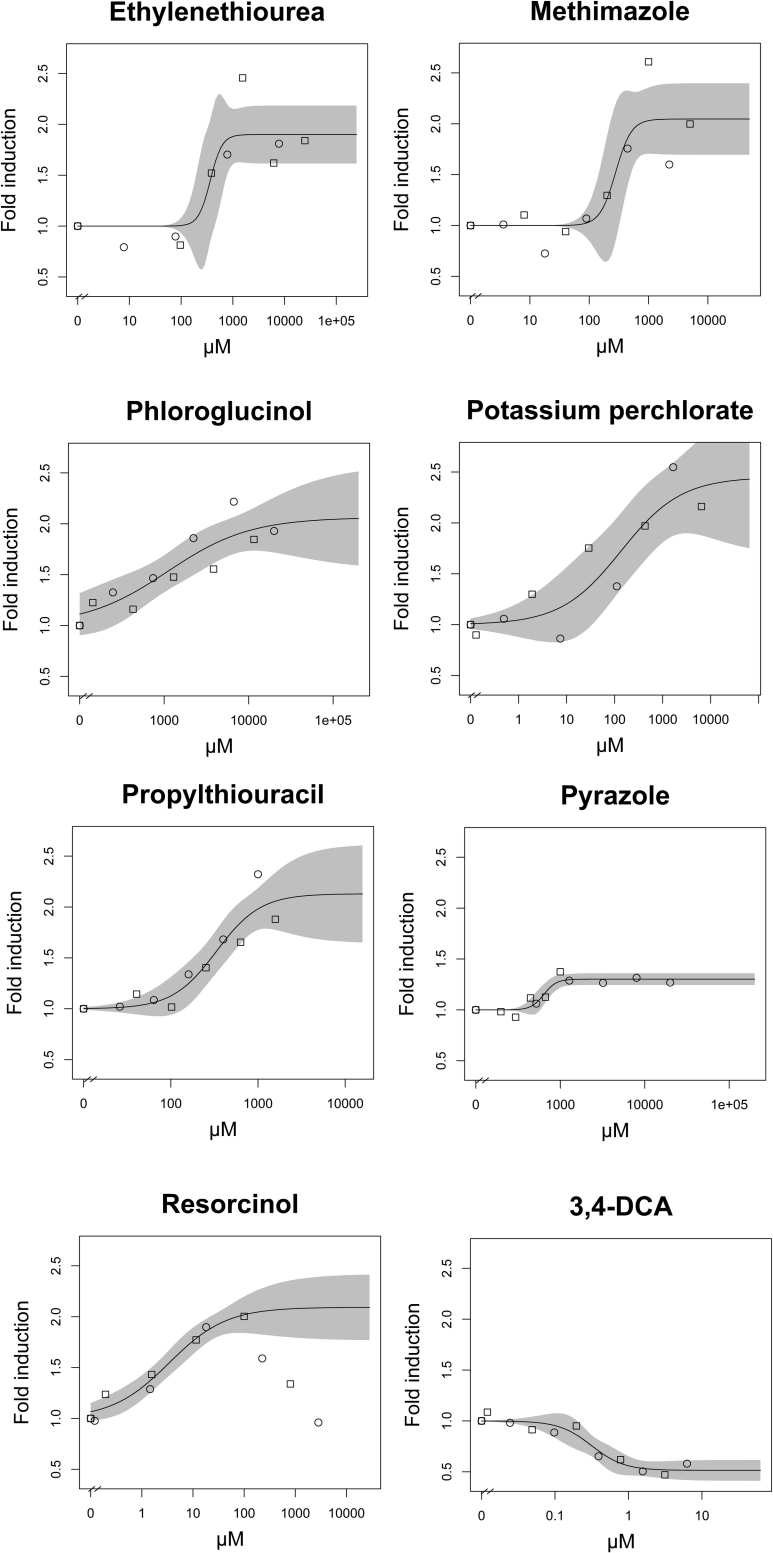
Concentration-response curves for induction of fluorescence in transgenic zebrafish embryos harboring a copy of the reporter gene mCherry and the regulatory region of thyroglobulin. Zebrafish embryos were exposed to known goitrogenic compounds and the negative control 3,4-dichloroaniline (3,4-DCA). The two different symbols represent two different replicates. Each replicate value represents the mean of at least 10 embryos analysed per concentration. The grey shaded zone represent 95% confidence intervals.

Goitrogenic effects were quantified by comparing the fluorescence in the thyroid glands of exposed embryos to controls and calculating the fold induction of mCherry fluorescence. Consistent with the negative feedback regulation of the HPT axis, all of the selected goitrogens provoked an induction of thyroglobulin expression after 3 days of exposure evident from the concentration-dependent increase of the tg:mCherry fluorescence (Fig 2, Table 1). At higher exposure concentrations, resorcinol showed a weaker induction or repression of fluorescence indicating a potential interfering or secondary toxic effect. A Hill slope model was used to derive EC50s and to compare the potency of compounds (Table 1). The Hill slope model may not be ideal since induction did not approach a clear equilibrium at maximum concentrations for some compounds. Therefore, a benchmark dose (BMD, [30]), i.e. a concentration at which a certain level of effect is reached, was calculated as well. We selected a 20% increase for the BMD calculation accounting for variability in the lower concentrations range (Table 1). The BMD can principally be calculated with diverse models. However, since the Hill slope model was describing the data appropriately in at the BMD20 level, no other model was considered. The negative control compound 3,4-DCA did not induce tg:mCherry fluorescence but led to a concentration dependent reduction in fluorescence. This may indicate a potential toxic interference with transcriptional activation of tg:mcherry. However, a specific interaction of 3,4-DCA cannot be excluded but need further research to understand the biological rationale for the observed inhibition. Weak evidence for a specific interaction is provided by a study with tadpoles that indicated elevated T3 level for exposure to 3,4-DCA and its parent compound diuron under certain conditions and at high concentrations [31]. This elevated T3 levels may result in a decreased expression of thyroglobulin via the HPT axis feedback loop. The tg:mCherry EC50 level of methimazole was slightly lower if compared to a previously obtained value (551 μM) obtained by a manual positioning and image analysis [22]. The potency based on the EC50s for induction levels lead to the following ranking of compounds for tg:mCherry induction: resorcinol < KClO_4_ < methimazole < 6-propyl-2-thiouracil < ethylenethiourea < pyrazole < phloroglucinol (Table 1). With the BMD20 a similar ranking was obtained, except that methimazole was slightly more potent than propylthiouracil and pyrazole was more potent than phloroglucinol. Maximum observed levels for tgmcherry ranged from 1.3 (pyrazole) to 2.5 potassium perchlorate. These differences may reflect differences in the efficacy of the modes of actions of the selected test compounds including thyroid cytotoxic compounds (pyrazole), sodium iodine symporter inhibitors (potassium perchlorate), and thyroid peroxidase inhibitors (ethylenthiourea, methimazole, propylthiourazile, phoroglucinol, resorcinol) as the most dominant group [13] or steric interactions with different enzyme inhibition efficacies.

Using the thyroid disrupting index (TDI = LC50 / EC50) as an indicator of potency a different ranking with resorcinol > phloroglucinol > KClO_4_ > ethylenethiourea > methimazole > pyrazole 6-propyl-2-thiouracil was obtained. Ranking based on effect concentrations was partially in agreement with other zebrafish-based goitrogen screening studies [13]. I.e. in both studies resorcinol and potassium perchlorate represented the compounds with the lowest effect concentration and phloroglucinol was indicated as the goitrogenic compound with the highest effect concentrations. Stronger differences were found when ranking was compared using the TDI. Particularly potassium perchlorate and resorcinol were differentially ranked (Table 1). These differences may have been caused as a result of different positions of the measured endpoint in the feedback loop of thyroid hormone regulation. While Thienpoint et al. [13] measured the reduction in T4 level directly, tg:mCherry fluorescence is induced as a result of a reduction in the T4 level via the feedback loop.

If compared to the sensitivity of *in vitro* assays, the zebrafish assays revealed goitrogenic effects at concentration 100–1000 fold higher [12]. The different sensitivity could be related to a higher metabolic capacity of fish embryo if compared to *in vitro* assays and/or compensation of the effects by the HPT axis.

### Relation of effect concentrations to hydrophobicity of test compounds

**Fig 3:**
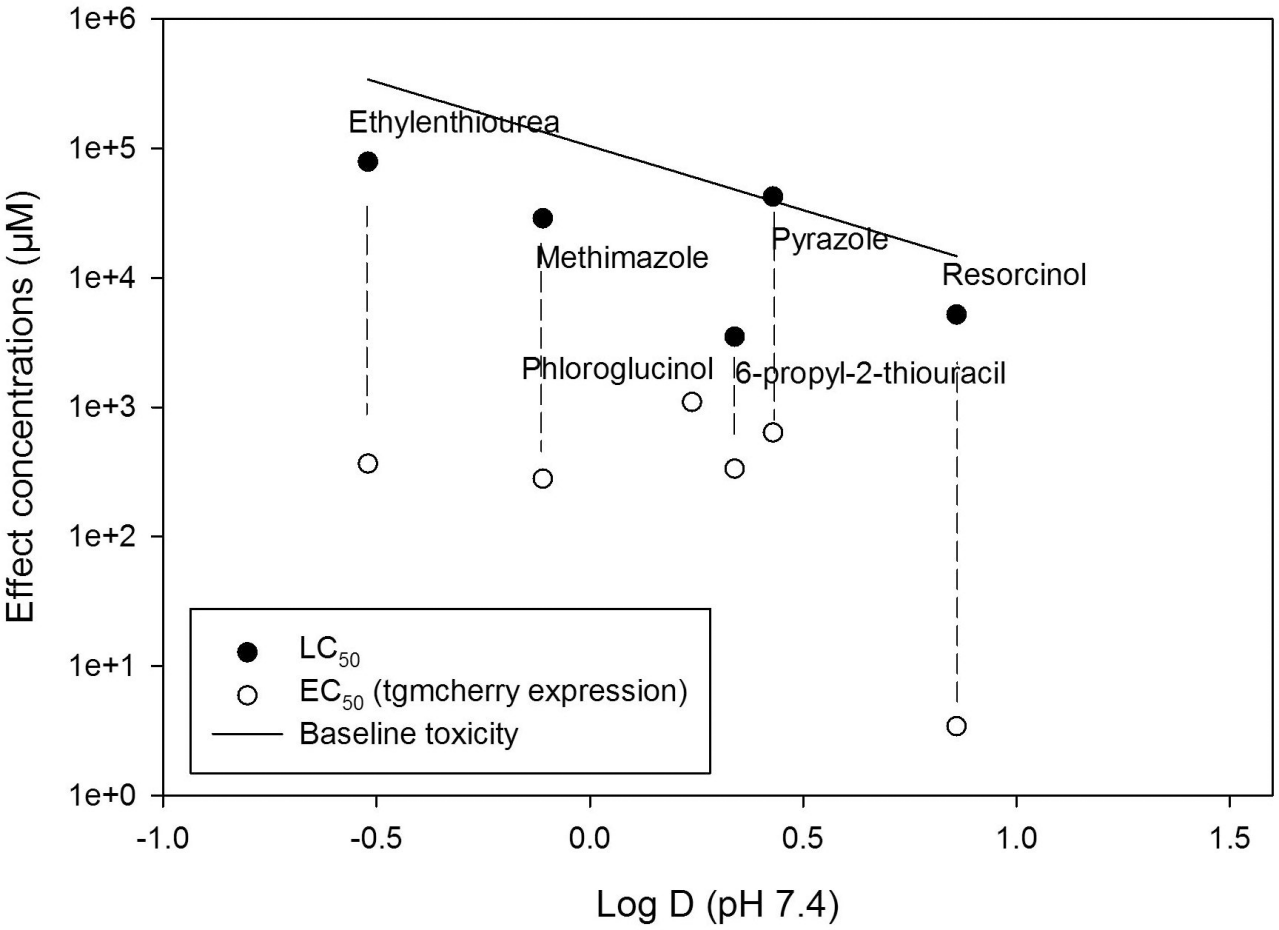
Effect concentrations for tg:mcherry induction and mortality in zebrafish embryo exposed from 48–120 hour post fertilization to known goitrogenic compounds. Effect concentrations are compared to the logD and the baseline toxicity (mortality) to indicate the specificity of the response. Note that the goitrogenic compound potassium perchlorate has not included in this figure given that the use of the logD is not applicable to inorganic compounds. Dashed lines connect the corresponding LC50 and EC50. No mortality was observed for phloroglucinol.

Given that goitrogenic effects are based on the interaction with specific proteins (e.g. sodium-iodide symporter and thyroperoxidase), it can be anticipated that the effect concentrations for goitrogenic effects are independent of hydrophobicity-driven baseline toxicity which is related to internal membrane concentrations that cause acute toxicity (mortality) in aquatic organisms (reviewed in e.g. [33]). Typically toxic ratios (ratio of calculated baseline versus observed LC50) of <10 are considered as indicators of unspecific baseline toxicity. In order to verify that the tg:mCherry induction was indeed not related to the hydrophobicity of a compound we compared the effect concentrations to those of the mortality and the log D of the chemicals. The log D was used instead of the log *K*_ow_ in order to correct for the (partial) ionization of some of the compounds at a given pH [34]. The LC_50_ values of most of the test compounds were within a factor of 10 of the baseline toxicity indicating that mortality was probably related to unspecific baseline toxicity (Fig 3). Only 6-propylthiouracil exhibited a slightly higher toxicity ratio (14). The LC50s were declining with increasing hydrophobicity providing further evidence that LC50 is driven to a large extent by baseline toxicity. In contrast no dependency on hydrophobicity was observed for the EC50 except that the lowest EC50 was observed for the most hydrophobic compound. However, the effect concentration were below 100fold of the baseline toxicity and the observed LC50 supporting the specificity of the tg:mCherry response.

### Conclusions

When using fluorescence imaging of transgenic fish embryos, a rate limiting step in the screening process is the manual orientation of fish embryos before the imaging. The latter can be supported by using appropriate agarose molds that are prepared using templates manufactured e.g. with a 3-D printer [35]. In contrast, the VAST BioImager platform used in this study provides an automated orientation based on pattern recognition algorithms [23]. The screening of tg(tg:mCherry) embryos is relatively slow with approximately 2 minutes required to analyze one embryo including loading, positioning, focusing and imaging. However, the imaging is processed unsupervised and offers the advantage that it can be combined with other phenotypic assessments from different positions. Our study has demonstrated that the combination of the VAST system, fluorescence imaging and concentration-response analysis represents an efficient method for the screening of compounds with goitrogenic activity. The automated method can easily be conducted by a user with little technical expertise. The method may not only be applied to identify individual potentially goitrogenic compounds but also be applied for the assessment of potential goitrogenic activities associated to environmental samples or cell/tissue extracts.

## Supporting information

S1 Fig Wavelength spectra of exposure solutions at EC50 concentrations for tgmcherry induction (ethylenthiorurea, methimazole, phoroglucionol, propylthiouracil, paryzole) or 2 mg/L (resorcinol). (PDF)

S2 Fig Concentration response curve for mortality in zebrafish embryo exposed from 72–120 hours post fertilization. (PDF)

S1 Table Raw data of phenotypes and mortality assessment. (XLSX)

S2 Table Settings of the VAST Bioimager. (PDF)

S3 Table Microscope settings. (PDF)

S4 Table Summary of thyroid gland normalised mcherry fluorescence. (XLSX)

S1 Workflow KNIME workflow for tgmcherry fluorescence analysis. (KNWF)
