# Supplementary material for: An automated screening method for detecting compounds with goitrogenic activity using transgenic zebrafish embryos
Source: PLoS One. 2018 Aug 29;13(8):e0203087. doi: 10.1371/journal.pone.0203087 (PMC6114901; doi:10.1371/journal.pone.0203087)
Supplement: S1 Fig — (PDF) [file pone.0203087.s001.pdf]

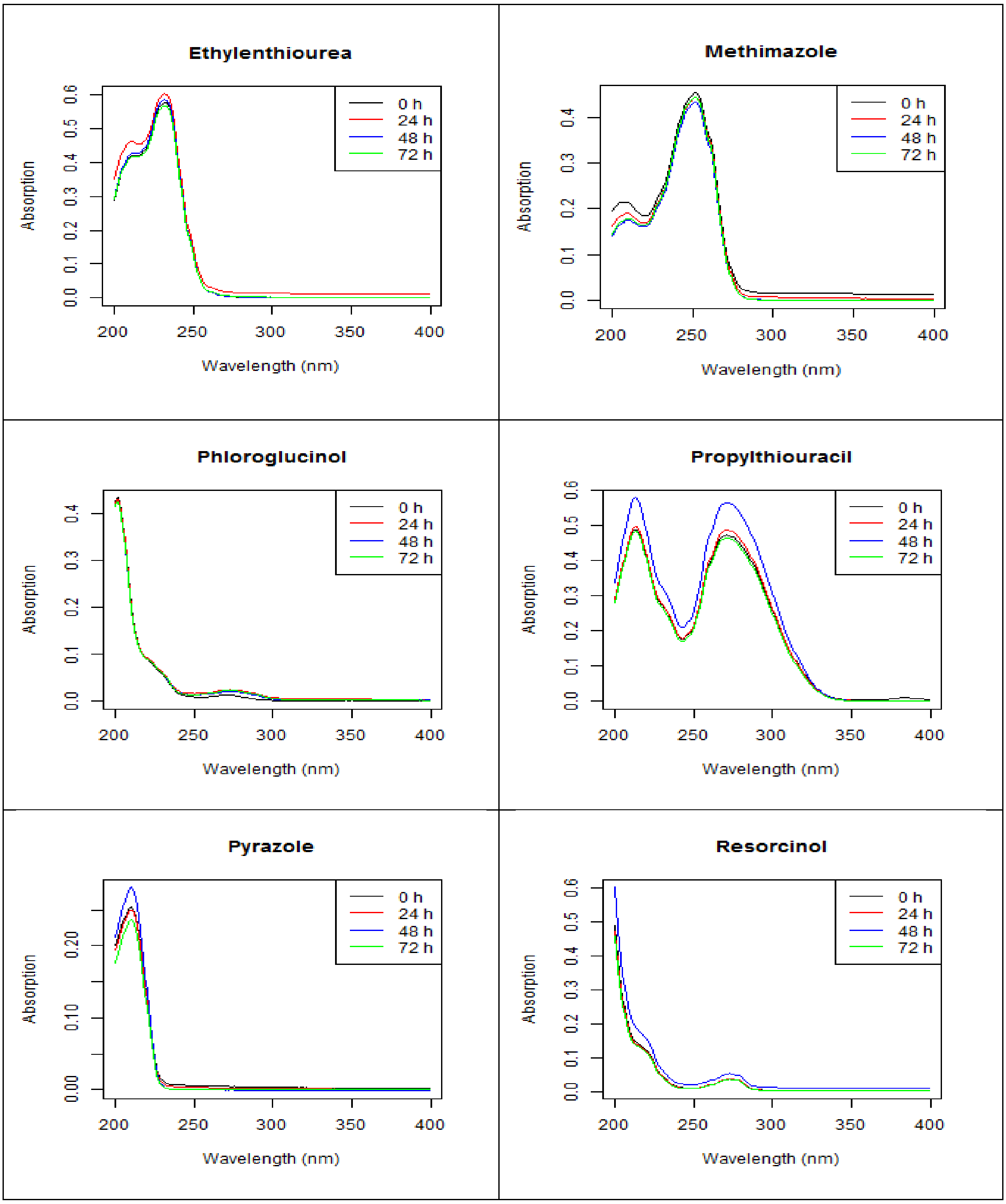

S1 Fig. Wavelength spectra of exposure solutions at EC50 concentrations for tgmcherry induction (ethylenthiorurea, methimazole, phoroglucionol, propylthiouracil, paryzole) or 2 mg/L (resorcinol). Exposure solutions were diluted 10fold except for phoroglucinol (100fold dilution) for recording of spectra at the start of the incubation (0 h) and at 24 h_intervals (24 h, 48 h, 72 h).
