# Supplementary material for: An automated screening method for detecting compounds with goitrogenic activity using transgenic zebrafish embryos
Source: PLoS One. 2018 Aug 29;13(8):e0203087. doi: 10.1371/journal.pone.0203087 (PMC6114901; doi:10.1371/journal.pone.0203087)
Supplement: S2 Fig — (PDF) [file pone.0203087.s002.pdf]

For figure legend see page 7

Compound/stage	Slope	LC10	LC50	RSS
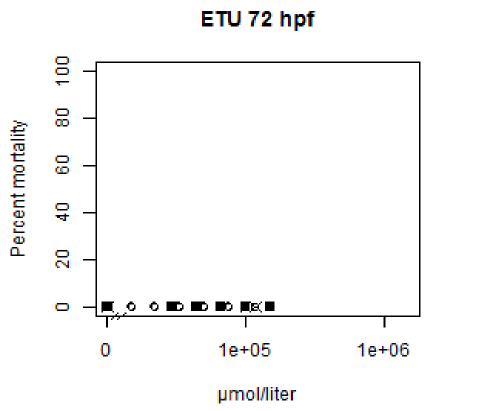	n/a	n/a	n/a	n/a
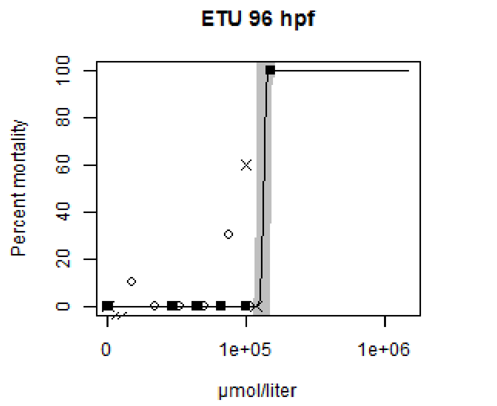	61.3	128406	133093	18.8
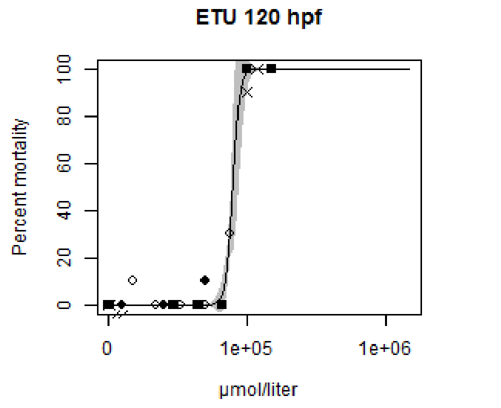	17.9	69818	78922	4.09

Compound/stage	Slope	LC10	LC50	RSS
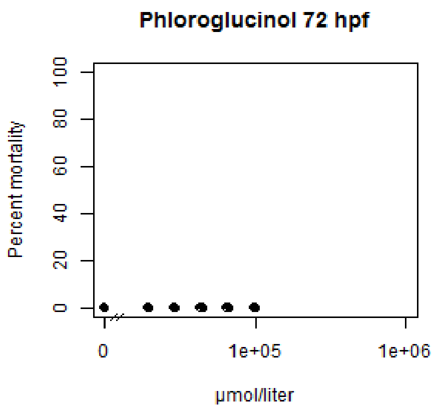	n/a	n/a	n/a	n/a
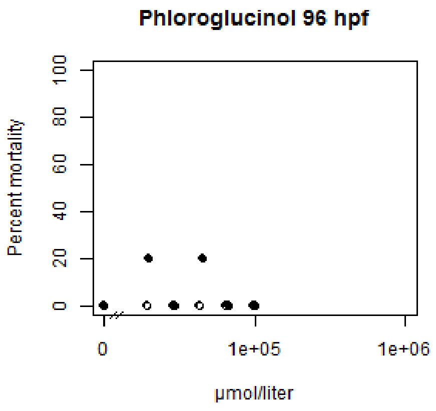	n/a	n/a	n/a	n/a
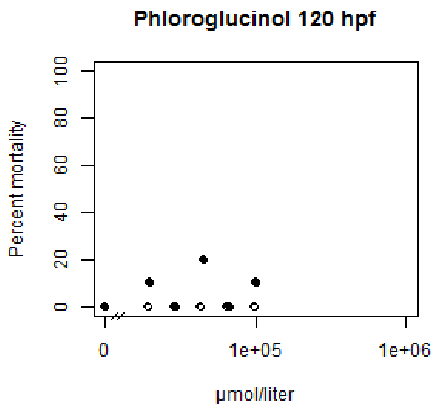	n/a	n/a	n/a	n/a

Compound/stage	Slope	LC10	LC50	RSS
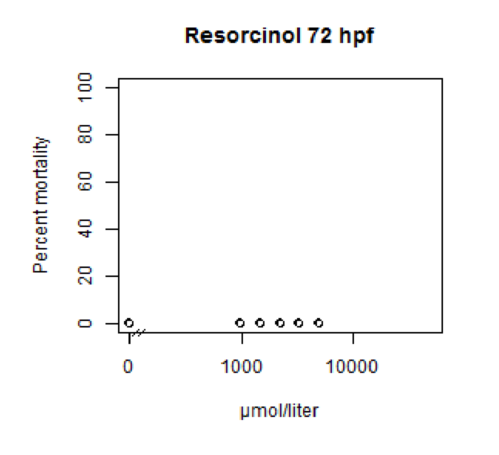	n/a	n/a	n/a	n/a
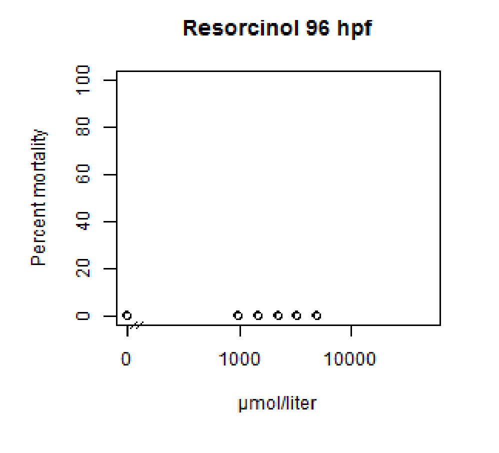	n/a	n/a	n/a	n/a
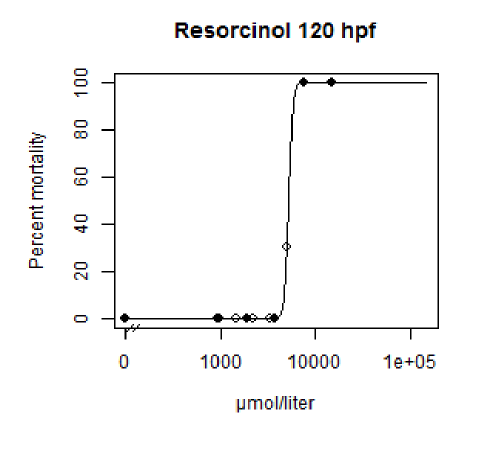	21.9	4701	5197	0.0271

Compound/stage	Slope	LC10	LC50	RSS
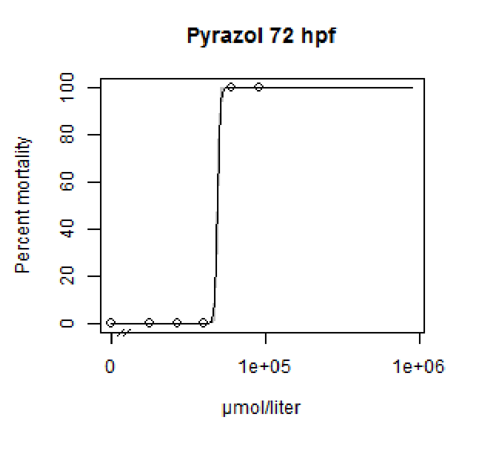	56.2	47074	48950	7.95E-04
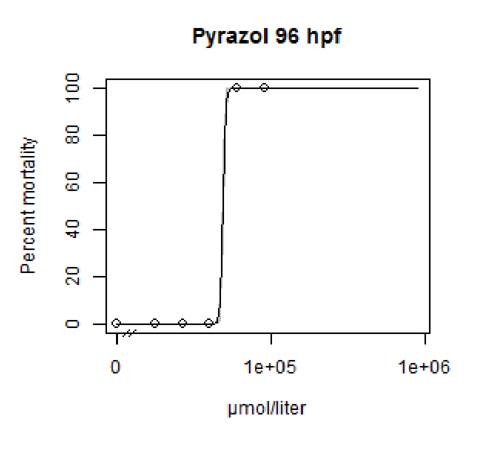	56.2	47074	48950	7.95E-04
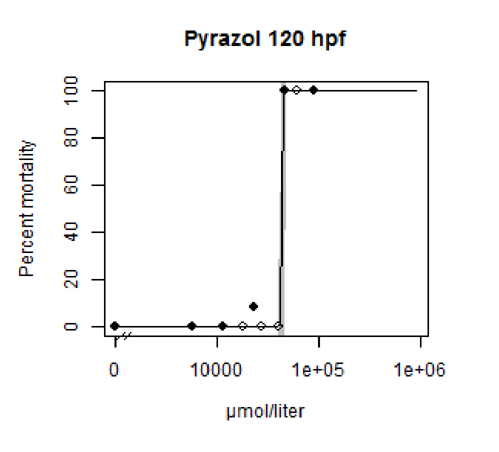	132	41727	42428	2.53

Compound/stage	Slope	LC10	LC50	RSS
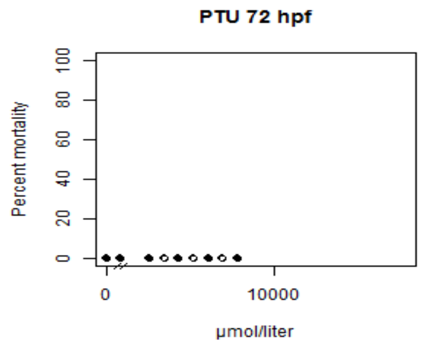	n/a	n/a	n/a	n/a
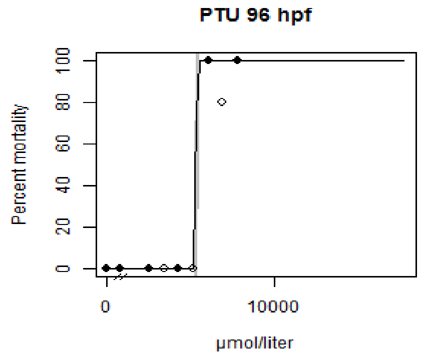	936	3458	3466	7.07
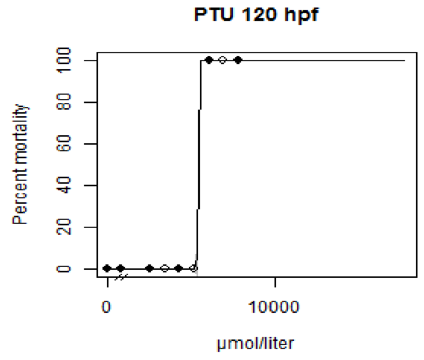	346	3478	3500	7.89E-08

Compound/stage	Slope	LC10	LC50	RSS
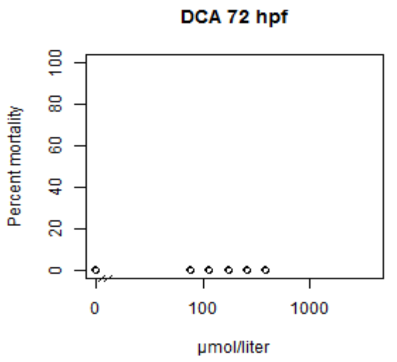	n/a	n/a	n/a	n/a
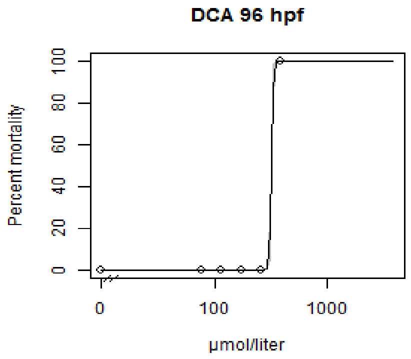	55.5	306	319	9.25E-04
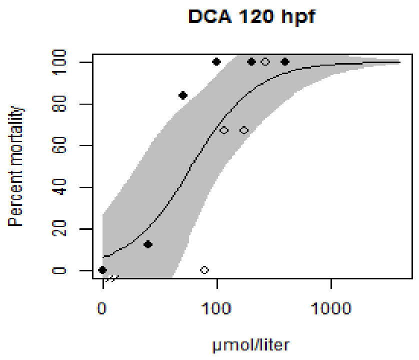	1.53	14.1	59.4	26.4

Compound/stage	Slope	LC10	LC50	RSS
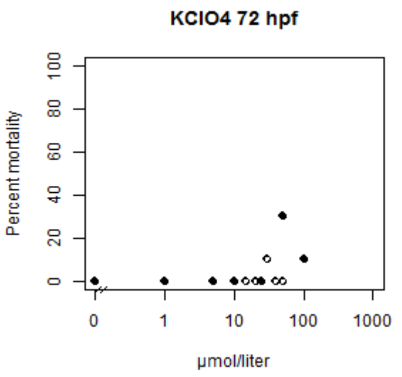	n/a	n/a	n/a	n/a
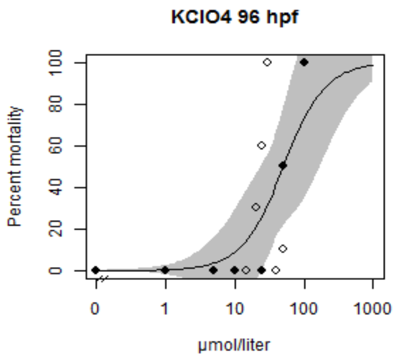	1.43	10862	50255	30.1
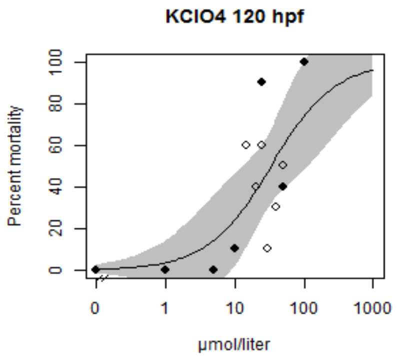	0.944	3232	33136	23.9

Fig. S2: Concentration response curve for mortality in zebrafish embryo exposed from 72-120 hours post fertilization. Mortality was analysed after 24, 48, and 72 h of exposure. LC10 and LC50s were obtained from modelled concentration response curves. RSS (residual sum of square) is used as an indicator of the goodness of the fit. Modelling is only conducted in case of a concentration dependent increase of mortality. DCA – 3,4-dichloroaniline, KClO4 – potassium perchlorate, PTU – propylthiouracile, ETU - ethylenethiourea
