# Supplementary material for: An automated screening method for detecting compounds with goitrogenic activity using transgenic zebrafish embryos
Source: PLoS One. 2018 Aug 29;13(8):e0203087. doi: 10.1371/journal.pone.0203087 (PMC6114901; doi:10.1371/journal.pone.0203087)
Supplement: S2 Table — (PDF) [file pone.0203087.s004.pdf]

S2 Table. Settings of the VAST Bioimager

Loading setup		Centering fish inside the capillary	
Max volume between flushes	300 µL	Displacement with 5 pump microsteps	1 mm
Initial pluger position	1000 µL	Min. visible fish length	3.9 mm
Limit for loading volume	1000 µL	Delay before and after adjusting position	700 ms
Attempt to re-load	off	Number of attempts	2
Re-loading backup volume	40 µL	**Backup**	
**Unloading setup (one fish at a time)**	Move forward after backup	Max backup volume	30 µL
Push trough volume	80 µL	Min backup volume	1 µL
Unloading volume 1	30 µL	Volume of a first backup pulse	2 steps
Push-back volume	40 µL	Move forward after backup	head first 7 microsteps, tail first 7 microsteps
Unloading volume 2	400 µL	**Object detection and rotation setup**	
Unload multiple fish in bulk	off	Drop of intensity threshold	20
**Operational mode setup**	Rotational angle	Min average intensity	130
Rotational position	Auto	Min intensity drop	25%
Hi-Resolution imaging	Auto	Bubbles and debris	detect and discard
Output		Manual	90
Bubbles and debries	Manual	Minimum similarity	0.60
**Auto mixer**	On	Rotational backlash	5 degrees
Speed	10%	**Pump speeds**	
Oscil	3 (sec)		
**Camera settings**		Loading speed	120
Exposure (Manual)	380 µs	Stepping speed	2
Gain (Manual)	1 dB	Backup speed	70
**White balance**		Unloading speed	2000
Red Gain	170%	Stepping speed	50
Blue Gain	200%	Flushing speed	4000
		Priming speed	2000
**Imaging with external device**		Wand aspirate speed	2000
X-position	290 µm	Backlash (microsteps)	14
Rotation angle	180		
Ctrl output	1		
Trigger output	1		
Bright field	off	**Software version**	1.2.5.1
Tray LED	off		
Trigger out delay	700 ms		
Trigger timeout	30000 ms		
Degree per rotation	360		
Rotation speed	30		
CSV file record	on		
