# Supplementary material for: An automated screening method for detecting compounds with goitrogenic activity using transgenic zebrafish embryos
Source: PLoS One. 2018 Aug 29;13(8):e0203087. doi: 10.1371/journal.pone.0203087 (PMC6114901; doi:10.1371/journal.pone.0203087)
Supplement: S3 Table — (PDF) [file pone.0203087.s005.pdf]

## S3 Table. Microscope settings

| | | | |
|---|---|---|---|
| **Excitation/Emmission wavelengths (nm)** | 515-560/590 LP | **Z-stack** | 9 images |
| **Exposure (fluorescence)** | 400 ms | **Z-size** | 300 µm |
| **Exposure (gain)** | 1 | | |
| **Illumination (FIM)** | 100 % | | |
| **Illumination (IL-FID)** | 6 | **Software** | |
| **Objective** | HCC Apo L U-U-I 20x/0.5 Water UV | **Leica application suite X** | Version 3.3.3.16958 |
